# The inducible *nitA* promoter provides a powerful molecular switch for transgene expression in *Volvox carteri*

**DOI:** 10.1186/s12896-015-0122-3

**Published:** 2015-02-18

**Authors:** Eva Laura von der Heyde, Benjamin Klein, Lars Abram, Armin Hallmann

**Affiliations:** Department of Cellular and Developmental Biology of Plants, University of Bielefeld, Universitätsstr. 25, D-33615 Bielefeld, Germany

**Keywords:** Ammonium, Co-transformation, *Gaussia princeps* luciferase gene, Genetic engineering, Green algae, Heterologous expression, Inducible promoter, Nitrate reductase, Nitrogen sources, Reporter genes, *Streptomyces rimosus aph*VIII gene, Volvocine algae

## Abstract

**Background:**

The multicellular green alga *Volvox carteri* represents an attractive model system to study various aspects of multicellularity like cellular differentiation, morphogenesis, epithelial folding and ECM biogenesis. However, functional and molecular analyses of such processes require a wide array of molecular tools for genetic engineering. So far there are only a limited number of molecular tools available in *Volvox*.

**Results:**

Here, we show that the promoter of the *V. carteri* nitrate reductase gene (*nitA*) is a powerful molecular switch for induction of transgene expression. Strong expression is triggered by simply changing the nitrogen source from ammonium to nitrate. We also show that the luciferase (*g-luc*) gene from the marine copepod *Gaussia princeps*, which previously was engineered to match the codon usage of the unicellular alga *Chlamydomonas reinhardtii*, is a suitable reporter gene in *V. carteri*. Emitted light of the chemiluminescent reaction can be easily detected and quantified with a luminometer. Long-term stability of inducible expression of the chimeric *nitA*/*g-luc* transgenes after stable nuclear transformation was demonstrated by transcription analysis and bioluminescence assays.

**Conclusion:**

Two novel molecular tools for genetic engineering of *Volvox* are now available: the nitrate-inducible *nitA* promoter of *V. carteri* and the codon-adapted luciferase reporter gene of *G. princeps*. These novel tools will be useful for future molecular research in *V. carteri*.

## Background

**Figure 1:**
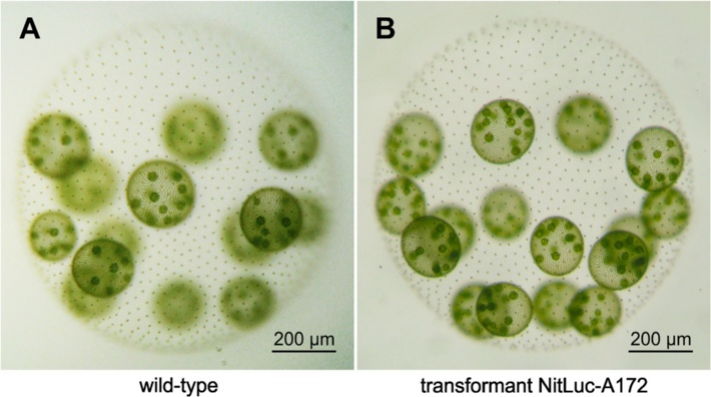
**The phenotype of** ***V. carteri*** **: wild-type and transformant.** *V. carteri* consists of ~2000 small, biflagellate, terminally differentiated, somatic cells at the surface and ~16 large reproductive cells in the interior of a spheroid. The reproductive cells develop inside the mother spheroid to embryos and later to juveniles. Up to 99% of the volume of such a spheroid consists of a complex, transparent, glycoprotein-rich extracellular matrix that holds all of the cells in place. **(A)** Wild-type spheroid of *Volvox carteri* f. *nagariensis* short before release of ~16 juvenile spheroids. **(B)** Spheroid of transformant NitLuc-A172 functionally expressing the *G. princeps* luciferase gene under control of the *V. carteri nitA* promoter. Transformant NitLuc-A172 shows the same phenotype as its wild-type parent.

The green alga *Volvox carteri* represents one of the simplest multicellular organisms: *Volvox* is composed of only two cell types, somatic and reproductive (Figure 1A). Despite its basic organizational concept, *Volvox* algae share many features that characterize the life cycles and developmental histories of complex multicellular organisms. Therefore, various aspects of multicellularity like cellular differentiation, morphogenesis, epithelial folding and ECM biogenesis have been studied in *Volvox* and its close relatives in the group of the volvocine algae [1,2]. Comparisons among volvocine algae open the possibility to discover universal rules that characterize the transition from unicellular organisms, such as *Chlamydomonas*, to complex, differentiated multicellular organisms like *Volvox* [1,2].

However, the technical feasibility of genetically manipulating a species of interest is a key factor for any detailed molecular analysis of gene or protein functions in order to reveal key aspects in development and evolution. A limited number of molecular tools and techniques are available in *Volvox* that provide a good basis for molecular manipulations: Stable nuclear transformation is possible [3], unselectable markers can be efficiently co-transformed together with selectable markers [3-5], some reporter genes and promoters are available [6-9], expression of chimeric genes works [6], foreign genes can be expressed [5,7], gene replacement by homologous recombination is feasible [10], plasmids or transposons can be used for gene tagging [11-13], and the genome of *V. carteri* has been sequenced [14].

Even if several promoters are available for genetic manipulations in *V. carteri*, there is a need for a strong, inducible promoter that can be switched on easily without affecting further development of the organism. The *hsp*70A promoter is able to increase expression in response to heat [8]; however, due to its permanent expression and the heat-inducibility of sexual development, it is unsuitable for use as a molecular switch. The arylsulfatase (*ars*) promoter is inducible by sulfur deprivation [6], but sulfur deprivation disturbs further development or even prevents it. Further inducible *Volvox* promoters have not been investigated so far.

We considered that the promoter of the endogenous nitrate reductase (*nitA*) might be suitable as a novel inducible promoter for genetic engineering in *V. carteri*. In plants, algae and fungi, nitrate assimilation genes including the nitrate reductase genes are expressed as a response to micromolar concentrations of nitrate and induction occurs within minutes [15-20]. Even if nitrate is the most commonly used nitrogen source because of its major abundance, the energy cost required for ammonium assimilation is lower than that of nitrate [21]. Therefore, ammonium provides a negative signal for nitrate assimilation, which leads to repression of nitrate transporters and enzymes of the pathway [16,19,20,22-27]. Also in *V. carteri,* accumulation of the nitrate reductase transcript has been shown to be both induced by nitrate and repressed by ammonium [4].

Aside from inducible promoters, reporter genes are required for genetic engineering. So far, the endogenous genes of arylsulfatase [6,28] and the sex inducer [6] as well as the heterologous genes of the codon-optimized green fluorescent protein (GFP) [29,30] and the hexose/H^+^ symporter HUP1 [7] have been presented as reporter genes in *Volvox*. Only the *ars* gene allows for easy quantification of the expression product and, therefore, is suitable for continuous use [6,28]. However, several suitable reporter genes are required in a model organism, e.g., for conducting multiplex assays. A good candidate for a new reporter gene in *V. carteri* seemed to be the luciferase gene (*g-luc*) of the deep sea copepod *Gaussia princeps* [31,32], which was previously adapted to the nuclear codon usage of *C. reinhardtii* [33]. The suitability of *g-luc* genes that were codon optimized for expression in the respective hosts has been shown in different organisms like human [34], the alga *Chlamydomonas reinhardtii* [33], the fungus pathogen *Candida albicans* [35] and in bacteria [36-38]. The G-Luc protein does not require any cofactor for activity and it catalyzes the oxidation of the substrate coelenterazine into the excited form of coelenteramide that releases a photon with a wavelength of 480 nm upon electron transition to the ground state [32,34]. G-Luc is the smallest (∼19.9 kDa) ATP-independent luciferase cloned to date [34,39] and it was shown to exhibit an activity up to 1,000-fold higher than *Renilla reniformis* luciferase, firefly luciferase and bacterial luciferases [34,37].

Here, we show that the codon-adapted luciferase gene of *G. princeps* is a suitable reporter gene in *V. carteri*. Emitted light of the chemiluminescent reaction can be easily detected and quantified with a luminometer; the chemiluminescence can also be detected on light-sensitive film, with a standard camera or even with the naked eye in the darkroom. We also demonstrate that the promoter of the *V. carteri* nitrate reductase gene (*nitA*) is a powerful molecular switch for induction of transgene expression. Strong expression is triggered by simply changing the nitrogen source from ammonium to nitrate. In addition, we provide proof of the long-term stability of inducible expression of the chimeric *nitA*/*g-luc* transgenes after stable nuclear transformation.

## Results

### Chimeric selectable marker and reporter genes for transformation

**Figure 2:**
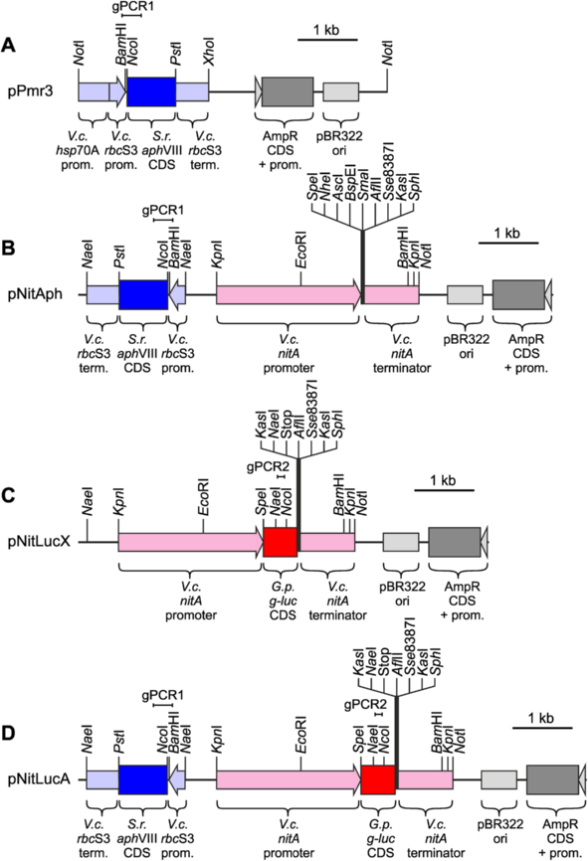
**Schematic diagram of the utilized selectable marker and reporter gene plasmids. (A)** Plasmid pPmr3 carries the selectable *S. rimosus aph*VIII gene driven by an artificial tandem promoter from the *hsp*70A and *rbc*S3 genes of *V. carteri* and a 3′-flanking sequence derived from the *rbc*S3 gene of *V. carteri* [8]. **(B)** Plasmid pNitAph contains the chimeric *aph*VIII selectable marker gene derived from plasmid pPmr3; however, the *aph*VIII gene is only driven by the *V. carteri rbc*S3 promoter (no tandem with *hsp*70A); it also contains the 3′-flanking sequence of the *V. carteri rbc*S3 gene. In addition, pNitAph contains the *V. carteri nitA* promoter region and also the 3′ flanking sequence of the *nitA* gene. An artificial multiple cloning site was introduced between the 5′ and 3′ control elements of *nitA* to allow for integration of a protein coding sequence of interest. **(C)** Plasmid pNitLucX contains the same *nitA* cassette as pNitAph (but no selectable marker); in addition, the *G. princeps g-luc* reporter gene was cloned into the multiple cloning site between the 5′ and 3′ control elements of *nitA*. **(D)** Plasmid pNitLucA is a derivative of pNitAph; the *G. princeps g-luc* reporter gene was inserted into the multiple cloning site. Amplified fragments of *rbc*S3/*aph*VIII (gPCR1) and *g-luc* (gPCR2) are indicated. *V.c.*, *Volvox carteri*; *S.r.*, *Streptomyces rimosus*; *G.p.*, *Gaussia princeps*; *g-luc*, luciferase gene.

For all transformation experiments, the *Streptomyces rimosus aph*VIII gene that confers resistance to paromomycin was used as a selectable marker [8]. The *aph*VIII gene was driven by strong *Volvox* promoters, i.e., either a tandem promoter made of both the *hsp*70A and the *rbc*S3 promoter (Figure 2A) or the promoter of *rbc*S3 alone (Figure 2B and D) [40,41]*.* For some experiments, this chimeric selectable marker gene was on a separate plasmid (pPmr3, Figure 2A) [8] and it was co-transformed with the reporter gene-carrying plasmid (pNitLucX, Figure 2C).

To allow for expression of a gene of interest under the control of the nitrate reductase (*nitA*) promoter of *V. carteri*, plasmid pNitX was constructed. This plasmid contains about 2.4 kb 5′ flanking sequence of the *nitA* gene [4] and about 0.9 kb of its 3′ flanking sequence. The latter sequence includes the terminator sequence of *nitA*. An artificial multiple cloning site with unique restriction sites was introduced between the 5′ and 3′ control elements to allow for integration of the protein coding sequence of interest. Plasmid pNitAph was derived from plasmid pNitX; it also carries the chimeric *aph*VIII selectable marker derived from plasmid pPmr3 (Figure 2A). Plasmids pNitAph and pNitX are universal starting plasmids for the construction of expression vectors for *Volvox*.

For analysis of the expression characteristics of the *nitA* promoter, the coding sequence of the luciferase (*g-luc*) from *Gaussia princeps* [31-33] was used as a reporter and, therefore, introduced into plasmids pNitX and pNitAph, respectively. The resulting plasmids pNitLucX and pNitLucA contain the *g-luc* coding sequence, which was previously adapted to the nuclear codon usage of *C. reinhardtii* [33], under control of the 5′ and 3′ control elements of the *nitA* gene of *V. carteri* (Figure 2C and D). The *Kas*I sites within the multiple cloning site and at the end of the *g-luc* coding sequence allow for DNA integrations to produce fusion proteins between the luciferase reporter and a certain protein of interest. Plasmid pNitLucX needs to be co-transformed with a selectable marker plasmid (pPmr3), whereas pNitLucA already contains the selectable marker *aph*VIII.

### Generation of transgenic *Volvox* strains using the *aph*VIII selectable marker

**Figure 3:**
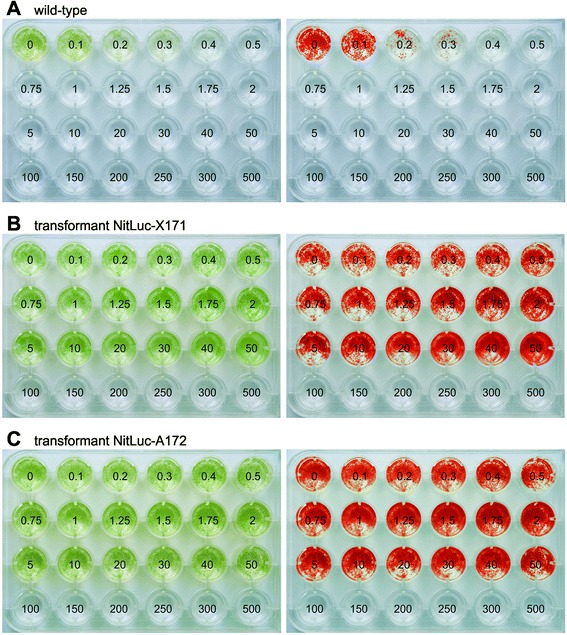
**Analysis of paromomycin resistance in wild-type and transgenic** ***V. carteri*** **strains.** For detailed analysis of resistance, identical quantities of *V. carteri* spheroids were exposed to increasing concentrations of paromomycin and incubated for six days. Numbers refer to the concentration of paromomycin [μg/ml] utilized. Natural color (left) and red-shifted, false-color images (right) are shown. **(A)** The wild-type *V. carteri* strain. **(B)** Transgenic *V. carteri* strain NitLuc-X171 that was co-transformed with pPmr3, the selectable marker plasmid, and plasmid pNitLucX, which carries the *g-luc* reporter gene driven by the *nitA* promoter. **(C)** Transgenic *V. carteri* strain NitLuc-A172 that was transformed only with plasmid pNitLucA, which carries both the selectable marker gene and the *g-luc* reporter gene driven by the *nitA* promoter.

To allow for selection of transformants with even weak transgene-mediated resistance, the lowest concentration of the antibiotic paromomycin that kills all wild-type *Volvox* cells was determined. To achieve this, a series of aliquots with the same number of wild-type *V. carteri* cells was exposed to increasing concentrations of paromomycin, incubated for six days, and screened for living (green) or dead (white) cells. The wild-type *Volvox* algae tolerated concentrations of up to 0.4 μg/ml paromomycin. A concentration of 0.5 μg paromomycin/ml or higher led to 100% cell death (Figure 3A). To allow for an objective and rapid discrimination between aliquots containing living cells and aliquots containing dead cells even in large-scale screenings of culture plates, red-shifted false-color images were created from standard photographs of the plates (Figure 3, right panel).

For transformation, a logarithmically growing culture of *V. carteri* was harvested by filtration and subjected to particle bombardment using DNA-coated gold microprojectiles as described before [9,40-42]. The unselectable plasmid pNitLucX (Figure 2C) was co-transformed with plasmid pPmr3 (Figure 2A), while the selectable plasmid pNitLucA (Figure 2D) was transformed alone.

After the transformation procedure, the algae were incubated in liquid medium and allowed to recover for three days before paromomycin was added. After 10–14 days of incubation, green and living spheroids were separated under microscopical control. The antibiotic resistance of separated spheroids was justified and concretized by incubation for six days in 2 ml *Volvox* medium with different concentrations of paromomycin ranging from 0.1 to 500 μg/ml (for concentrations see Figure 3). Transformants with the most robust resistance tolerated concentrations up to 50 μg/ml paromomycin (Figure 3B and C). Thus, *Volvox* transformants exhibited an up to 125-fold higher tolerance against paromomycin than the wild-type strains.

### Genomic integration of chimeric gene constructs

PCR was used to verify the stable integration of the chimeric genes into the genome of *V. carteri* transformants. To this end, genomic DNA was isolated from transformants that were able to grow for three weeks under selective pressure by exposure to paromomycin. Using *aph*VIII-specific primers and the isolated genomic DNA as a template, PCR fragments of the expected size were obtained from seventeen transformants. The transformants were also checked for the presence of the *g-luc* reporter gene using *g-luc* specific primers. Fifteen of the seventeen transformants were shown to contain both the *aph*VIII gene and the *g-luc* gene. Obviously, two transformants contained only the intact *aph*VIII gene but no intact *g-luc* gene or no *g-luc* gene at all. Therefore, both clones were excluded from further investigations. The parent wild-type strain did not yield any *aph*VIII or *g-luc* specific products, as expected.

The transformation efficiency was calculated to be at about 2.7 · 10^−5^, which is quite similar to previously reported transformation rates of about 2.5 · 10^−5^ [3] and about 10^−6^ [5]. The co-transformation rate was quite high, but the total number of transformants was too low to allow for a significant statistical analysis. However, the total number of obtained transformant clones was almost the same, regardless of whether the selectable marker gene and the reporter gene were on two different plasmids or both genes were on the same plasmid.

### Expression of the *g-luc* reporter gene in transformants

All transformants that contained both intact *aph*VIII and *g-luc* genes were assayed for functional expression of luciferase. Luciferase activity was assayed using the bioluminescent substrate coelenterazine and a MiniLumat luminometer. PCR-positive transformants clearly showed luciferase activity in contrast to the parent wild-type strain. However, the expression rate varied between the different transformants. The expression of the luciferase was analyzed in detail after a long-term incubation of the transformants without selective pressure (see below).

### Long term stability of DNA integration and gene expression

For any biotechnological approach with transgenic organisms, the stability and unimpaired expression of the transgenes for a long period of time is of fundamental importance. Therefore, the genomic integration and expression of the *aph*VIII and *g-luc* genes was re-examined after more than one year of cultivation of the *V. carteri* transformants or, in other words, after more than 250 generations. Cultivation was without selective pressure during this period.

**Figure 4:**
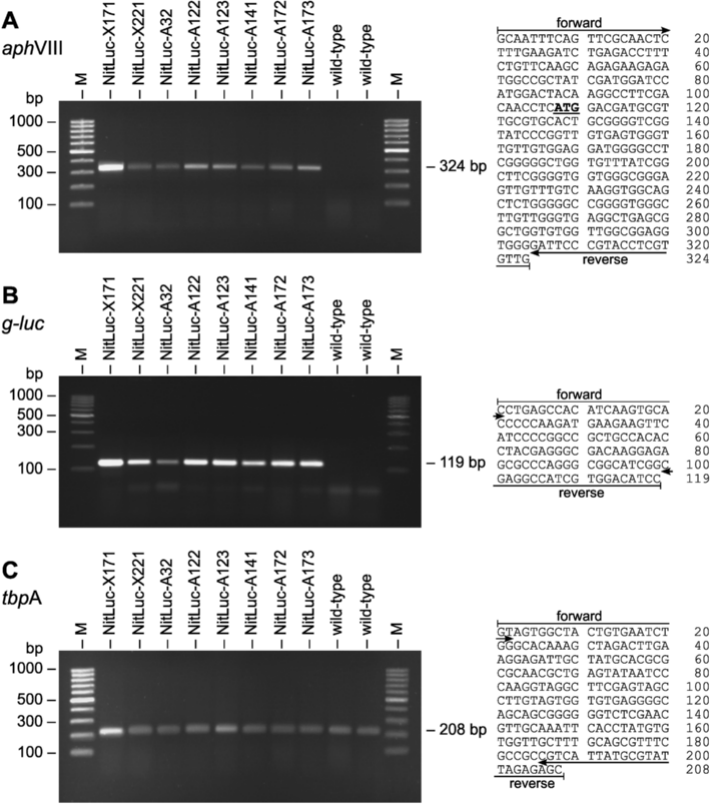
**Proof of genomic integration of both** ***aph*** **VIII and** ***g-luc*** **genes in transformants. (A)** Paromomycin resistant transformants and the parent wild-type strain were analyzed for presence of the *aph*VIII gene in the genome by genomic PCR. The expected size of the PCR fragment produced in transformants was 324 bp (gPCR1, Figure 2A and D). **(B)** Genomic PCR analysis of paromomycin resistant transformants and the parent wild-type strain for the presence of the *g-luc* gene in the genome. The expected size of the PCR fragment produced in transformants was 119 bp (gPCR2, Figure 2C and D). **(C)** As a control, a 208 bp fragment of an unaffected *Volvox* gene (*tbp*A) was amplified both from all transformants and from wild-type strains just as expected. **(A-C)** M-lanes refer to the molecular weight marker. PCR fragments were cloned and sequenced. The obtained sequences are given right of each gel image. The positions of the primers and the start codon (underlined, bold) are indicated. This PCR assay was carried out more than 250 generations after transformation of the strains.

If the DNA of the chimeric *aph*VIII gene (coming from plasmids pPmr3 or pNitLucA) was present in the genome, a 324 bp chimeric fragment, containing 107 bp of the *rbc*S3 promoter region and 217 bp of the *aph*VIII coding region, was expected to be amplified by PCR using the corresponding specific primers (Figure 2A and D, gPCR1). The results are shown in Figure 4A. Transformants were also re-investigated for the presence of the *g-luc* reporter gene (Figure 4B). If the DNA of the former plasmids pNitLucX or pNitLucA was present in the genome, a fragment of 119 bp was expected to be amplified with the corresponding specific primers (Figure 2C and D, gPCR2). In both experiments (*aph*VIII and *g-luc*) the amplified fragments were cloned and sequenced (Figure 4A, and B, right side). The sequences were just as anticipated. In both cases, the parent wild-type strain did not yield any product, as expected (Figure 4A, and B). As a further control, a 208 bp fragment of an unaffected *Volvox* gene (*tbp*A) was amplified both from all transformants and from wild-type strains, just as expected (Figure 4C). Thus, it can be concluded that the genomic integrations of both the chimeric *aph*VIII and *g-luc* genes are still present even more than 250 generations after transformation. Therefore, the genomic integrations can be referred to as stable.

We also re-examined the functional expression of the chimeric *aph*VIII and *g-luc* genes by antibiotic resistance and luciferase assays, respectively. All transformants exhibited about the same antibiotic resistance and luciferase activity compared to the values determined right after transformation. Thus, not only the genomic integration but also the expression of the chimeric *aph*VIII and *g-luc* genes is stable in *V. carteri*.

### Inducibility of the *nitA* promoter-driven *g-luc* reporter gene in transformants

**Figure 5:**
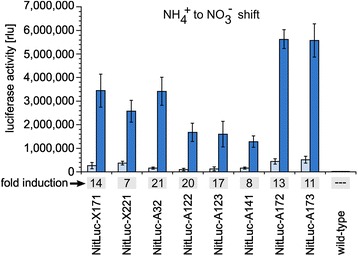
**Quantification and inducibility of luciferase activity in transformants.** Transformants expressing the *g-luc* reporter gene under control of the *nitA* promoter and the parent wild-type strain were investigated for luciferase activity. The diagram shows the measured enzyme activity in untreated specimen (left column) and specimen induced by a switch from NH_4_^+^ to NO_3_^−^ (right column). The columns represent the mean of three independent experiments. The standard deviation is indicated. The factor of induction is given underneath the columns. The luciferase activity of each sample was normalized with its chlorophyll concentration. This luciferase activity assay was carried out more than 250 generations after transformation of the strains.

All transformants were assayed for inducible expression of luciferase. For it, transformants and wild-type cultures were grown in standard *Volvox* medium with ammonium. After a media shift to *Volvox* medium with nitrate, incubation was continued for 1 h under standard conditions. Cell extracts were obtained from all strains before and after incubation in nitrate medium. The cell extracts were checked for luciferase activity using a luminometer (Figure 5). To allow for quantitative comparison of activity measurement results, all samples were normalized with the respective chlorophyll content. The results clearly show that luciferase expression is inducible by the NH_4_^+^ to NO_3_^−^ switch.

In transformants, the expression increased by a factor of 7 to 21 after the switch to nitrate. The average induction factor was 13.9 +/− 5.2. Except for the different number of obtained transformants, we couldn’t see any principal differences between transformants generated with pNitLucX and pPmr3 or with pNitLucA alone.

**Figure 6:**
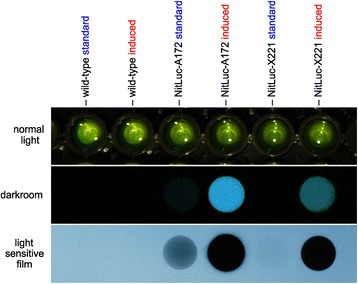
**Visualization of nitrate-inducible luciferase activity.** Parental wild-type spheroids and transformants expressing the *g-luc* reporter gene under control of the *nitA* promoter (NitLuc-A172 and NitLuc-X221) were investigated for light emission as a result of luciferase activity both under standard conditions (standard) and after induction by a switch from NH_4_^+^ to NO_3_^−^ (induced). Upper row: standard photo showing the assay setup. Middle row: photo (30 s exposure) without any extraneous light after addition of the coelenterazine substrate. Lower row: exposure to a light-sensitive film for 10 min after addition of the coelenterazine substrate.

For later large scale applications, we also visualized the expression and inducibility of luciferase in a more direct way. Although transformants show a wild type phenotype under the light microscope (Figure 1), a glow is visible even to the naked eye in the darkroom when the coelenterazine substrate is added to cell lysates of NO_3_^−^-induced transformants (Figure 6). Exposure to a light-sensitive film also allows for a simple, semi-quantitative determination of chemiluminescence of many transformants in parallel (Figure 6).

### mRNA and protein expression kinetics after induction of the *nitA* promoter-driven chimeric reporter gene

**Figure 7:**
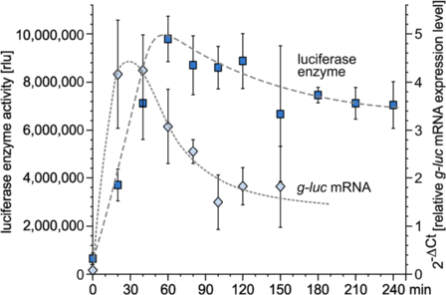
**mRNA and protein expression kinetics after nitrate-induction of the** ***nitA*** **promoter.** Transformant NitLuc-A172 expressing the *g-luc* reporter gene under control of the *nitA* promoter was induced by a switch from NH_4_^+^ to NO_3_^−^ medium at 0 min. Both the *g-luc* mRNA and the luciferase activity were investigated at several time points after induction. The relative *g-luc* mRNA level was calculated in comparison to the reference gene *tbp*A using the 2^-ΔCt^ method for analysis of the real-time RT-PCR results. The protein expression was determined with luciferase activity assays using the chlorophyll concentration of each sample for normalization.

The characteristics of the *nitA* promoter were analyzed after induction of the *nitA* promoter-driven chimeric *g-luc* reporter gene with a switch from NH_4_^+^ to NO_3_^−^. Taking transformant NitLuc-A172 (Figure 1B) as an example, both the *g-luc* mRNA and the luciferase activity were investigated at several time points from 0 to 240 min after the switch to NO_3_^−^ (Figure 7). For it, total RNA was isolated and the *g-luc* mRNA expression level was analyzed using quantitative real-time RT-PCR. The luciferase enzyme activity was quantified in parallel using the chlorophyll content as a reference.

After the switch to NO_3_^−^, the *g-luc* mRNA expression increases quickly and peaks at about 30 min. In the following 2 hours, the expression level decreases slowly and reaches a plateau at about 30% of the maximal expression. The luciferase enzyme activity shows a similar profile but the maximum is reached about 1 hour after the switch, which corresponds to a delay of 30 min in relation to the mRNA peak. The luciferase activity reaches about 10 million relative light units (rlu) at the peak (Figure 7). The luciferase expression level decreases even slower than the mRNA expression level and reaches a plateau at about 7 million rlu or about 70% of the maximal expression, which might reflect that the luciferase protein turnover is much lower than the turnover of its mRNA.

## Discussion

The codon-adapted luciferase gene of *G. princeps* has proved to be a suitable reporter gene in *V. carteri*, even if the codon-optimization has been done for *Chlamydomonas* [33]. Although *Volvox* diverged from unicellular ancestors like *Chlamydomonas* at least 200 million years ago and codon usage became different from each other [43], the relationship obviously is still close enough to allow for good expression in *Volvox*.

The assay for luciferase activity through detection of emitted light of the chemiluminescent reaction proved to be very effective in *Volvox* just as in several other organisms before [33-38]. The assay is quick and easy, enzyme activity can be quantified reliably and the measurement is both robust and well reproducible. The assay also exhibits high sensitivity because even a small amount of enzyme can catalytically process many substrate molecules to produce a detectable signal. Accurate quantification is done with a luminometer. In large scale analyses, light detection on light-sensitive films or with a camera allows for qualitative assessments. Since *Gaussia* luciferase reaction is quite fast [34] it is important to measure the emitted light immediately after coelenterazine injection.

Previous work with the *Gaussia* luciferase showed that this enzyme is very stable even in body fluids such as blood and urine [44], in cell culture medium [34] and in cell extracts [33]. In our standard assay, we measured the enzyme activity in crude cell extracts obtained by ultrasonic treatment. No further purification of the enzyme was necessary. In preliminary experiments we even measured the luciferase activity in *Volvox* cultures containing intact spheroids (data not shown). The activity values obtained with intact spheroids were almost as high as the activity values obtained with cell extracts. Therefore, in high-throughput screenings, direct measurement of cell cultures without ultrasonic treatment might be an even easier alternative, but reproducibility of the results needs be checked before. The functionality of the assay in cell cultures without ultrasonic treatment is attributed to the high cell-membrane permeability of coelenterazine.

Altogether, the codon-adapted luciferase reporter gene outshines previous reporter genes used in *Volvox* in terms of its sensitivity, robustness and ease of use.

In addition to the *g-luc* reporter gene, the promoter of the nitrate reductase gene [4] turned out to be a powerful molecular tool for genetic engineering in *V. carteri*. Two of our vectors (pNitX and pNitAph) allow for *nitA*-driven expression of any target gene after a single cloning step. The *nitA* promoter drives expression of the gene of interest by simply changing the nitrogen source from ammonium to nitrate [4,45], all the other components of the *Volvox* medium remain unchanged throughout. The change of media is easily managed with *Volvox* because the spheroids can be quickly collected on a nylon screen and transferred to the new medium. Changing the nitrogen source disturbs growth and development, if at all, only to a minimum in contrast to other methods for promoter induction using chemicals [46], hormones [47-51] or increased temperatures (heat shock promoters) [40-42,45]. The switching time of the *nitA* promoter is very short: After induction by changing the nitrogen source, mRNA expression increases immediately and reaches its maximum about 30 min after induction (Figure 7), which is similar to results in earlier work with the complete unmodified endogenous *nitA* gene [4]. The expression and induction characteristics caused by our *nitA* fragment demonstrate, that the *nitA* promoter is fully working and all required cis-elements are included. Therefore, our constructs also might be interesting for those who want to analyze this promoter and its regulation in more detail.

A further advantage of using the *nitA* promoter for genetic engineering is that the expression rate of the target gene is not only influenced by nitrate but also by ammonium. As mentioned, ammonium prevents the uptake of nitrate [19,27] and nitrate is required for the induction of the *nitA* promoter. Thus, submicromolar amounts of ammonium added together with the normal amounts of nitrate might allow for adjustment of the expression rate of the target gene in a given transformant. It also might be possible to switch off expression by adding larger amounts of ammonium.

## Conclusion

Two new molecular tools are now available for genetic engineering of the green alga *Volvox carteri.* The *nitA* promoter of *V. carteri* allows for inducible expression of target genes and induction occurs immediately after changing the nitrogen source from ammonium to nitrate. The change of the nitrogen source is easily managed and it disturbs neither growth nor development. The codon-adapted luciferase gene of *G. princeps* turned out to be a powerful reporter gene in *Volvox*. The assay for luciferase activity is easy, robust, reproducible and sensible; moreover, it works in crude cell extracts.

## Methods

### Strains and culture conditions

The wild-type *Volvox carteri* f. *nagariensis* strains Eve10 (female) and Isanuma *Volvox* 5 (male) originate from Japan and have been described previously [52-54]. Cultures were grown in modified *Volvox* medium [55] with 1 mM ammonium chloride (NH_4_Cl) as a nitrogen source and 0.5 mM calcium chloride (CaCl_2_) as a calcium source. For induction of the *nitA* promoter, the nitrogen source was switched from NH_4_^+^ to NO_3_^−^, i.e., the *Volvox* medium with 1 mM NH_4_Cl and 0.5 mM CaCl_2_ was replaced by *Volvox* medium with 0.5 mM calcium nitrate [Ca(NO_3_)_2_] both as a nitrogen and a calcium source. Thus, the final nitrogen concentration was 1 mM before and after the switch from NH_4_^+^ to NO_3_^−^ and the calcium concentration remained at 0.5 mM throughout. For changing the medium, the *Volvox* spheroids were collected on a 100 μm mesh nylon screen, washed on the screen with NO_3_^−^ medium and then transferred to the NO_3_^−^ medium. Cultures were grown at 28°C in a cycle of 8 h dark/16 h cool fluorescent white light [56] at an average of ~100 μmol photons m^-2^ s^-1^ photosynthetically active radiation (PAR) in glass tubes, Fernbach flasks or 10 l glass bottles. The glass tubes had caps that allow for gas exchange, and the Fernbach flasks and 10 l glass bottles were aerated with approximately 50 cm^3^ sterile air/min.

### Transformation vectors

For construction of plasmids containing parts of the *V. carteri nitA* gene, i.e., plasmids pNitX, pNitAph (Figure 2B), pNitLucX (Figure 2C) and pNitLucA (Figure 2D), the promoter and terminator regions of *nitA* were amplified by recombinant PCR using genomic DNA of *V. carteri* as a template. A DNA fragment that covers the promoter region of the *nitA* gene, beginning at the ATG start codon and ending 2.4 kb upstream of the start codon, was amplified using the sense primer 5′-ggtaccTCAGGTGCTGATCGAAGCAC and the antisense primer 5′-Gcccggg*TCCGGA*ggcgcgcc*GCTAGC*actagtCACGAAAATGTTTCAGATGC. The sense primer introduced a *Kpn*I site (lower case) and the antisense primer introduced the first part of an artificial multiple cloning site containing the sites *Sma*I, *Bsp*EI, *Asc*I, *Nhe*I and *Spe*I (lower case or italics). A DNA fragment that covers the terminator region of the *nitA* gene, beginning at the stop codon and ending 0.9 kb downstream of the stop codon, was amplified using the sense primer 5′-gcggccgcGACAGCATAATCGTTACAAG and the antisense primer 5′-acccggg*CTTAAG*cctgcagg*GGCGCC*gcatgcCGATCCGACTCTCGGAGGTTAAC. The sense primer introduced a *Not*I site (lower case) and the antisense primer introduced the second part of the artificial multiple cloning site containing the sites *Sma*I, *Afl*II, *Sse*8387I (including *Pst*I), *KasI* and *Sph*I (lower case or italics). Both amplified PCR fragments were cloned by blunt end ligation. Then the promoter fragment was cut out from the vector with *Kpn*I and *Sma*I and the terminator fragment was cut out with *Sma*I and *Not*I. Both fragments were ligated with each other via *Sma*I and simultaneously with a *Kpn*I-*Not*I digested pBluescriptII SK(−) vector (multiple fragments cloning). The resulting plasmid, pNitX, is 6.3 kb in size.

Plasmid pPmr3 (Figure 2A) with the *Streptomyces rimosus aph*VIII selectable marker gene has been described previously [GenBank: AY429514] [8,40-42]. For integration of the selectable marker into plasmid pNitX, the resistance cassette containing the *V. carteri rbc*S3 promoter region (277 bp), the *S. rimosus aph*VIII coding region (831 bp) and the *V. carteri rbc*S3 terminator region (543 bp), was amplified by recombinant PCR using the primers 5′-gccggcATCAAATCTGCGCGTCAGAG and 5′-gccggcGTTCCCCGTGTGAGGCCTTG. Plasmid pPmr3 (Figure 2A) was used as a template. The primers introduced *Nae*I restriction sites (lower case) on both ends of the 1.6 kb DNA fragment. The PCR fragment was cloned by blunt end ligation, cut out with *Nae*I and cloned into the unique *Nae*I site of plasmid pNitX. The resulting plasmid pNitAph (Figure 2B) is 7.9 kb in size.

For analysis of the expression characteristics of the *nitA* promoter, the coding sequence of the luciferase from *Gaussia princeps* was used as a reporter. It was previously engineered to match the codon usage in *C. reinhardtii* and was available on plasmid pPsaD-GLuc [33]. The luciferase coding region (570 bp) was amplified by recombinant PCR using the sense primer 5′-actagt*ATG*GTCAACGGCGTGAAGGTG and the antisense primer 5′-cttaag*TTA*CGTATCGTCGCCGCCGG (start and stop codons in italics). The sense primer introduced a *Spe*I restriction site (lower case) and the antisense primer introduced an *Afl*II restriction site (lower case). These sites allow for cloning into the unique *Spe*I and *Afl*II restriction sites of the multiple cloning sites in pNitX and pNitAph (Figure 2B). The resulting plasmids are pNitLucX (Figure 2C), which is 6.8 kb in size, and pNitLucA (Figure 2D), which is 8.4 kb in size.

### Coating of microprojectiles

Gold microprojectiles (1.0 μm in diameter, Bio-Rad, Hercules, CA) were coated with the required plasmids as previously described [40,41]. The DNA-coated microprojectiles were resuspended in 60 μl EtOH and kept at 4°C for use within 3 h.

### Stable nuclear transformation by particle bombardment

About 8,000 wild-type spheroids were collected on a 40 μm stainless steel mesh and used as a target. Transformation was performed using a Biolistic® PDS-1000/He (Bio-Rad) particle gun. One-sixth of the DNA-coated microprojectiles were evenly spread on a macrocarrier (Bio-Rad) that was placed in a macrocarrier holder (Bio-Rad). The transformation procedure was as previously described [9,40-42]. The burst pressure of the rupture disks (Bio-Rad) was 1,100 psi, the rupture disk-macrocarrier distance was adjusted to 7 mm, the macrocarrier-stopping screen distance was 8 mm, the stopping screen-target cell distance was adjusted to 11 cm and the bombardment chamber was evacuated to 28 inch Hg. After the bombardment step, the algae were washed on the steel mesh. The bombardment procedure was repeated five times. Then, the algae were washed off from the steel mesh and evenly distributed among 15 to 25 Erlenmeyer flasks (150 ml size) filled with ~40 ml *Volvox* liquid medium. To select for paromomycin resistance in transgenic algae, 3 μg paromomycin per ml (paromomycin sulfate, Sigma-Aldrich, USA) was added to the medium at the third day and an additional 3 μg paromomycin/ml at the sixth day after transformation. From the fifth day on after the first paromomycin addition, bombarded cultures were examined by dark-field stereomicroscopy (MZ16A; Leica; Germany) and each green alga in a background of bleached spheroids was transferred into a microtiter well of a 24-well culture plate containing *Volvox* medium with 0.5 μg paromomycin/ml. After incubation in the presence of the antibiotic for at least six days, the microtiter wells were inspected for green and living transformants.

### Paromomycin-resistance assay

Cells of transformed or wild-type *V. carteri* strains were checked for their resistance to paromomycin as previously described [42] using a wide range of paromomycin concentrations from 0 to 500 μg/ml (Figure 3).

### Primer design

Oligonucleotide primers were designed using the primer analysis software OligoCalc [57] and Primer-BLAST [58].

### Isolation of genomic DNA

The extraction of genomic DNA was according to Edwards et al. [59] with minor modifications. The purity and quantity of the DNA was checked using agarose gel electrophoresis and a NanoDrop™ 1000 (Thermo Scientific) UV/Vis spectrophotometer.

### Genomic PCR

PCR reactions with genomic DNA as a template were carried out as previously described [40-42] using a gradient PCR thermal cycler (Mastercycler Gradient; Eppendorf, Germany). The PCR products were cloned and sequenced.

### Isolation of total RNA

Approximately 250 μl of concentrated, frozen algae were grinded with a mortar and a pestle and total RNA was extracted using 1 ml of phenol-based TRI Reagent (Sigma-Aldrich, St. Louis, MO) and 300 μl trichloromethane. RNA precipitation and RNA purification was as previously described [40,41,60].

### Quantitative Real-Time RT-PCR

The SensiFAST SYBR Hi-Rox One-Step Kit (Bioline) and a CFX96 Touch™ Real-Time PCR Detection System (Bio-Rad) were used for real-time RNA quantification. All real-time RT-PCR experiments were carried out in triplicates. The products of all real-time RT-PCR reactions were visualized using agarose gel electrophoresis to assure amplification of a single product of the correct size. The specific primers were as follows: 5′-CCTGAGCCACATCAAGTGCAC and 5′-GGATGTCCACGATGGCCTCG for amplification of a fragment of the luciferase (*g-luc*) transcript from *G. princeps* (codon-optimized for *C. reinhardtii*) [33] (expected cDNA length: 119 bp), and 5′-GTAGTGGCTACTGTGAATCTGG and 5′-GCTCTCTAATACGCATAATGACG for amplification of a fragment of the TATA-box binding protein transcript *tbp*A [61] (expected cDNA length: 118 bp), which has been established as a reference gene for quantitative gene expression studies in *V. carteri* [62]. The cycling conditions in the CFX96 Touch™ Real-Time PCR Detection System were as follows: reverse transcription was at 50°C for 30 min followed by polymerase activation at 95°C for 2 min and 40 cycles of DNA amplification with 95°C for 5 s, 55°C for 10 s and 72°C for 5 s. Melting curves were recorded to check for amplification of a single specific product. The relative expression level was calculated using the 2^-ΔCt^ method [63,64].

### Luciferase assays

For quantification of luciferase activity, cultures with 10 to 40 spheroids per ml were split into two aliquots of unequal volumes (200 ml and remaining volume) and the algae of each aliquot were collected on a 100 μm mesh nylon screen. For each strain, the smaller aliquot was assayed as a reference (see below) and the larger aliquot was transferred into *Volvox* medium with nitrate for induction of the *nitA* promoter. The culture was incubated under standard conditions for the respective times. Then, at the different points in time, 200-ml aliquots of the algae were harvested. If many clones were checked for enzyme inducibility at the same time, only one point in time was analyzed (1 h after induction; Figure 5), whereas up to 10 points in time were analyzed for the protein expression kinetics (Figure 7). The harvested algae were resuspended in 1 ml of assay buffer and disrupted by four 15 s pulses of direct sonication using a Sonopuls™ HD2070 sonicator (Bandelin Electronic, Berlin, Germany) at 70% power with breaks of 15 s on ice. The resulting lysates were frozen immediately in liquid nitrogen and stored at −70°C for not longer than 24 h before use. The lysates were thawed on ice (in the dark) and centrifuged at 20,000 g for 5 min (at 4°C). 20 μl of the supernatant were mixed with 125 μl assay buffer and incubated for 15 min at 20°C. 50 μl of 0,01 mM coelenterazine (Fluka, Neu-Ulm, Germany) in assay buffer were added and quantification of luciferase activity was conducted as described previously [33] using a MiniLumat LB9506 luminometer (Berthold). Luminescence was recorded as relative light units (rlu). Induction factors were calculated by comparison of samples from NO_3_^−^-induced cultures versus cultures that were kept in standard medium with NH_4_^+^. The luciferase activity of each sample was set in relation to its chlorophyll concentration that is proportional to the cell density (see “Chlorophyll determination” below).

For assays on light-sensitive films and for photo-optical documentation of luciferase activity, algae were collected, resuspended in assay buffer, frozen, thawed and centrifuged as described above. A volume of 200 μl lysate was mixed with 50 μl 0.02 mM coelenterazine and the enzyme reactions were performed in a 96-well micro-assay plate (chimney well, black with clear bottom; Greiner Bio-One, Solingen, Germany) positioned on a chemiluminescence-sensitive film (Retina XBA; Fotochemische Werke, Berlin, Germany) for 10 min at 35°C [65]. In addition, the luciferase activity was documented photo-optically using a Nikon D200 photo camera (Nikon, Tokio, Japan) with exposure times of 30 s.

### Chlorophyll determination

Because exact spheroid or cell densities of *Volvox* cultures can hardly be measured, the chlorophyll concentration of cell lysates was determined and used for normalization of the samples used for luciferase assays. For it, 200 μl cell lysate was mixed with 800 μl acetone and incubated at 20°C for 45 min (in the dark). The suspension was then centrifuged at 16,000 g for 5 min and the absorption of the supernatant was measured at 647 nm and 664 nm in an UV/Vis spectrophotometer (Ultrospec 2100 Pro UV/Vis Spectrophotometer, GE Healthcare, Uppsala, Sweden). The chlorophyll content was determined according to Jeffrey and Humphrey [66].

### mRNA and protein expression kinetics

For generation of mRNA and protein expression kinetics after induction of the *nitA* promoter-driven chimeric reporter gene, transformant NitLuc-A172 was grown synchronously in 10 liter flasks with *Volvox* medium containing ammonium chloride. A sample of uninduced algae was taken as a reference (as described above) and the remaining culture was washed (5 h before onset of the dark phase in the cycle of 8 h dark and 16 h light) and transferred to *Volvox* medium containing nitrate instead of ammonium chloride. Samples with volumes of 400 ml (RNA isolation) and 200 ml (enzyme assay, see paragraph “Luciferase assays” above) were taken at several points in time (Figure 7). The concentrated algae were stored at −70°C for RNA isolation or preparation of cell lysates for luciferase activity measurements, respectively.

## Abbreviations

*aph*VIII: *S. rimosus* aminoglycoside 3′-phosphotransferase VIII gene
*g-luc*: *G. princeps* luciferase gene (adapted to *C. reinhardtii* nuclear codon usage)
*hsp*70A: heat shock protein 70A gene
*nitA*: nitrate reductase gene A
PCR: Polymerase chain reaction
*rbc*S3: ribulose bisphosphate carboxylase small chain gene 3
rlu: relative light units
RT: Reverse transcription
*tbp*A: TATA-box binding protein gene A
